# Rationally Designed DNA Nanostructures for Drug Delivery

**DOI:** 10.3389/fchem.2020.00751

**Published:** 2020-09-24

**Authors:** Fan Xu, Qing Xia, Pengfei Wang

**Affiliations:** Institute of Molecular Medicine, Shanghai Key Laboratory for Nucleic Acid Chemistry and Nanomedicine, State Key Laboratory of Oncogenes and Related Genes, Department of Oncology, School of Medicine, Renji Hospital, Shanghai Jiao Tong University, Shanghai, China

**Keywords:** drug deliver systems, DNA self-assembly, nanocarrier, biological barrier, rational design

## Abstract

DNA is an excellent biological material that has received growing attention in the field of nanotechnology due to its unique capability for precisely engineering materials via sequence specific interactions. Self-assembled DNA nanostructures of prescribed physicochemical properties have demonstrated potent drug delivery efficiency *in vitro* and *in vivo*. By using various conjugation techniques, DNA nanostructures may be precisely integrated with a large diversity of functional moieties, such as targeting ligands, proteins, and inorganic nanoparticles, to enrich their functionalities and to enhance their performance. In this review, we start with introducing strategies on constructing DNA nanostructures. We then summarize the biological barriers ahead of drug delivery using DNA nanostructures, followed by introducing existing rational solutions to overcome these biological barriers. Lastly, we discuss challenges and opportunities for DNA nanostructures toward real applications in clinical settings.

## Introduction

Nanocarriers capable of the potent delivery of therapeutic molecules play a pivotal role in enhancing their therapeutic efficacy in clinic, and typically aid in minimizing systemic toxicity, improving biostability/bioavailability, and strengthening delivery efficiency to targeted tissues, cells, or subcellular locations (Savjani et al., 2012; Huang et al., 2013; Din et al., 2017; Gustafson et al., 2018; Zhang R. et al., 2019). Enormous progress has been made in this area, accompanied by the evolution of a large diversity of nanomaterials. Several delivery systems, such as liposomes and cationic dendritic polymers, have been approved for clinical use (Cheng et al., 2011; Bulbake et al., 2017). Meanwhile, many other types of delivery systems, including inorganic particles and cell mimics, are under extensive study in laboratories or in clinics (Chen et al., 2016; Pang et al., 2017). Nevertheless, there are many limitations that remain to be tackled in order to fully realize the therapeutic potential of drug delivery systems, which include but are not limited to acute toxicity in the short term and unknown toxicity in the long run (Lv et al., 2006), heterogenicity of formulated delivery systems (Adjei et al., 2014), and very limited targeting delivery efficiency (Wilhelm et al., 2016).

With the substantial development of DNA molecular self-assembly in the last four decades, DNA-based nanocarriers have emerged as promising delivery systems for drug delivery. Derived from the unique Watson-Crick base pairing, it is fully possible to create a variety of DNA nanostructures with a well-defined size and homogeneous geometry through the sequence design of DNA molecules followed by a straightforward self-assembly process, which is highly predictable, reproducible, and scalable (Seeman and Sleiman, 2017). DNA is a natural biological molecule, which is biodegradable with minimal toxicity. Furthermore, drugs like DNA intercalators (e.g., doxorubicin) and nucleic acids (e.g., siRNA, antisense oligonucleotides) can be easily integrated into DNA-based nanocarriers (Li et al., 2011; Lee H. et al., 2012; Zhao et al., 2012; Fakhoury et al., 2014; Rahman et al., 2017). Aided by well-established nucleic acid synthesis and bioconjugation techniques, DNA strands may be incorporated with various functional moieties to enrich the functionality of delivery systems, such as loading a variety of macromolecules (e.g., protein, inorganic particle) or targeting ligands (Li et al., 2019; Shin et al., 2020). These unique properties make DNA-based nanomaterials an attractive drug delivery system. After introducing responsive components, DNA nanocarriers can acquire dynamic capabilities in response to a variety of physiological or non-physiological stimuli (Zhang Y. et al., 2019). However, the high complexity of *in vivo* microenvironments poses great challenges to their proper performance in living organisms (Zhao Z. et al., 2019). Therefore, rational design strategies are essential in order to maintain their multifunctional delivery properties *in vivo*. With improved understanding of their *in vivo* fate, the drug delivery performance of DNA nanocarriers could then be highly predictable and designable (Jiang et al., 2019). In this review, we will first introduce the design methods for fabricating DNA-based nanocarriers, and then discuss strategies of tuning DNA-based nanocarriers for overcoming biological barriers to maximize their potential for efficient drug delivery.

## Constructing DNA Nanostructures for Drug Delivery

### DNA Self-Assembly Strategies

**Figure 1:**
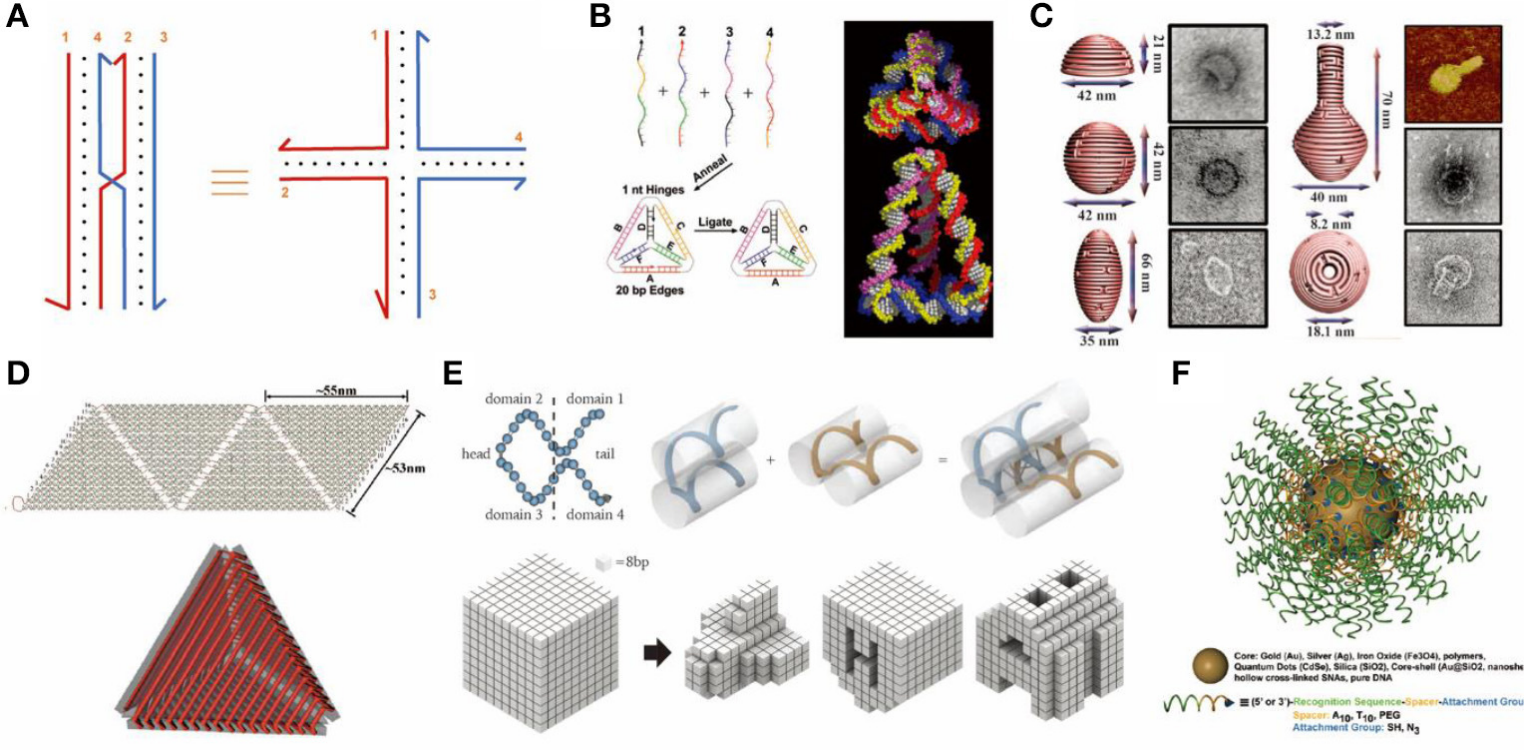
Rationally designed DNA nanostructures. **(A)** An immobile Holliday junction. **(B)** DNA tetrahedron formed from 4 single-stranded DNAs. Adapted with permission from Goodman et al. (2005). Copyright 2005 AAAS. **(C)** 3D DNA origami structure with precisely controlled curvature. Adapted with permission from Han et al. (2011). Copyright 2011 AAAS. **(D)** DNA container constructed by folding and joining single-layered 2D origami sheets. Adapted with permission from Ke et al. (2009b). Copyright 2009 ACS. **(E)** DNA objects assembled from single-stranded tiles (or DNA bricks). Adapted with permission from Ke et al. (2012). Copyright 2012 AAAS. **(F)** Spherical nucleic acids tethered onto a gold nanoparticle core. Adapted with permission from Cutler et al. (2012). Copyright 2012 ACS.

Distinguishing from DNA vectors like plasmids, DNA nanocarriers are based on the *de novo* design of self-assembled DNA nanostructures. Invented by Seeman in the early 1980s, methods of constructing DNA nanostructures have advanced rapidly in the last four decades (Seeman, 2020). In the early stage of DNA nanotechnology, by breaking the sequence symmetry of the Holliday junction, the original slidable 4-arm branched DNA junction can be fixed and serves as a basic assembling unit to build higher-order structures (Figure 1A) (Seeman, 1982). Seeman's group later introduced a tile-based method for DNA assembly. They bundled DNA strands by DNA crossovers to form a variety of units called “tiles.” Repeated tiles were interconnected by complementary single-stranded extensions (sticky ends) and eventually grew into large assemblies (Ding et al., 2004). Following the initial immobile 4-arm Holliday junction, junctions of a diverse number of arms were designed as DNA building blocks (Ma et al., 1986; Wang et al., 1991; Wang and Seeman, 2007). Well-designed tiles allow for the construction of highly-ordered 1D, 2D, and 3D DNA structures. Mao and Seeman later reported the successful fabrication of DNA 3D crystals by using tensegrity triangular tiles (Zheng et al., 2009). Yan and Laban first designed a point-star-like tile containing bulged T loops. These loops are located in the center of the tile linking two adjacent arms (Yan et al., 2003). Further tuning the length of the loops allows their linking arms to bend from the original geometric plane with varying degrees. For instance, 3-point star tiles with varied lengths of loops can self-assemble into a number of different polyhedra including tetrahedrons, dodecahedrons, and Buckyball structures (He et al., 2008; Wang et al., 2016).

Differing from polyhedrons assembled by point-star-tiles which contain repeated units of tiles, DNA nano-objects such as cubes, tetrahedrons, and octahedrons may also be assembled from multiple single-stranded DNAs (Chen and Seeman, 1991; Zhang and Seeman, 1994; Goodman et al., 2005). For example, in 2005, Goodman et al. reported a simple and rapid way to assemble 4 single-stranded DNAs into tetrahedrons by one annealing step with a high yield up to 95%, which has been widely used for a variety of applications (Figure 1B) (Goodman et al., 2005).

Another breakthrough in the field of DNA nanotechnology was the invention of the DNA origami technique by Rothemund (2006), where one long single-stranded scaffold DNA is folded by a set of complementary short strands (staples) to form various 2D objects (Rothemund, 2006; Wang et al., 2017). Further, Shih and colleagues extended this method to 3D by pleating the plane of DNA helixes and arranging them into compact lattices (Douglas et al., 2009; Ke et al., 2009a). Yan and colleagues built 3D hollow objects by programming the curvature of designated helixes via adding or deleting bases within DNA crossovers (Figure 1C) (Dietz et al., 2009). 3D origami structures may also be assembled by folding and joining single-layered 2D origami sheets, which resulted in the successful generation of container-like DNA boxes (Figure 1D) (Andersen et al., 2009; Ke et al., 2009b).

In 2012, Yin and colleagues reported a simple single-stranded tile (SST) or DNA brick method to build 2D and 3D objects of arbitrary shapes (Ke et al., 2012; Wei et al., 2012; Ong et al., 2017). SST consists of 4 domains that interact with adjacent tiles. Each SST can bind to four adjacent counterparts by complementary domains and assemble into prescribed structures (Figure 1E) (Ke et al., 2012).

### Other Strategies to Build DNA Nanostructures

Jones et al. used inorganic nanoparticles to provide rigidity and initiated another branch of DNA nanotechnology (Jones et al., 2015). For example, single-stranded DNA oligonucleotides with thiol modified ends can tightly bind to Au nanoparticles. This type of structure was named “spherical nucleic acids” (SNA, Figure 1F) (Cutler et al., 2012). These DNA-particle hybrids have a variety of applications, including drug delivery. Particularly, it has been used for the combination of gene regulation and photothermal therapy (Kim J. et al., 2016).

Rolling-circle amplification (RCA) is another popular method for constructing drug delivery DNA nanostructures, which employ DNA/RNA polymerase to generate large quantities of long concatemeric DNA products from a predesigned circular DNA template (Mohsen and Kool, 2016). The products contain repeated sequences and can be cleaved to generate functional DNA fragments. Due to its high yield of products, the nucleic acids can directly condense into particles with or without the help of condensing agents (Lee J. B. et al., 2012), which offers a great advantage toward high-volume drug loading.

## Enhancing Biostability and Extending Circulation Time

Rapid and non-specific clearance is a great challenge for nanoparticle-based drug delivery systems *in vivo*. After intravenous administration, a sufficient circulation time of nanocarriers is a prerequisite for good therapeutic efficacy (Wang et al., 2013). It is generally challenging for DNA nanocarriers to reside in physiological environments due to the following reasons. Firstly, as a biological material, DNA is prone to degradation by deoxyribonucleases (DNases) in serum. Secondly, the ionic strength of body fluids is quite different from the DNA assembly buffers. In particular, low cationic concentration may cause the disassembly of DNA nanostructures due to increased electrostatic repulsion between negatively charged DNA helices (Hahn et al., 2014). Thirdly, the opsonization effect by non-specific adsorption of serum proteins induces macrophages to engulf DNA nanocarriers for clearance (Surana et al., 2013). Lastly, DNA nanostructures have a high tendency for fast renal or hepatic clearance (Messaoudi et al., 2019).

There are numerous strategies to address the challenges that DNA nanostructures face. One is from a design point of view. For instance, it has been reported that less compact DNA structures or structures with a wireframe geometry exhibit higher resistance to nuclease degradation or cation-depletion-induced structure disassembly (Jiang et al., 2016; Kielar et al., 2018; Chandrasekaran et al., 2020). Apart from this design perspective, many other strategies are available to enhance the biostability of DNA nanostructures which will be introduced in detail in the following section.

### DNA Backbone Modification

**Figure 2:**
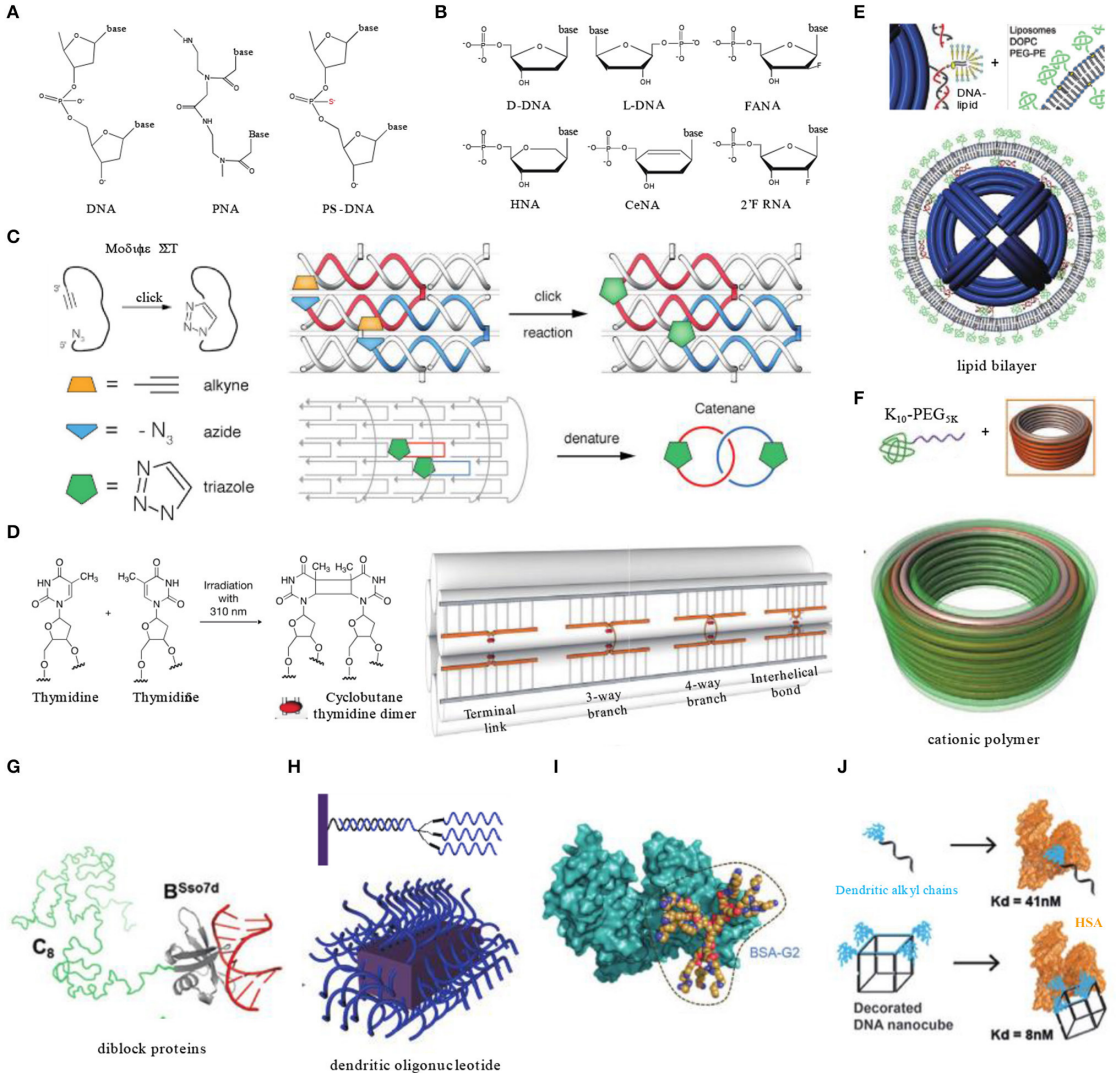
Enhancing the biostability and extending the circulation time of DNA nanocarriers. **(A)** Natural DNA backbone and common inter-nucleotide linkage modifications involved in DNA nanotechnology. **(B)** Natural nucleosides and their analogs involved in DNA nanotechnology. **(C)** Covalent crosslinking of 6-Helix DNA structure containing strands modified with azides and alkynes by click chemistry. Adapted with permission from Cassinelli et al. (2015). Copyright 2015 Wiley. **(D)** Light triggered dimer formation of adjacent thymidine residues in DNA origami objects. Adapted with permission from Gerling et al. (2018). Copyright 2018 AAAS. **(E)** Lipid bilayer encapsulation of DNA nanocages. Adapted with permission from Perrault and Shih (2014). Copyright 2014 ACS. **(F)** Electrostatically coating DNA origami nanostructures with polyamine-PEG block copolymers. Adapted with permission from Ponnuswamy et al. (2017). Copyright 2017 Springer Nature. **(G)** A diblock protein containing a non-sequence-specific DNA binding domain (B^Sso7d^) and an unstructured blocking domain (C_8_) for DNA structure coating. Adapted with permission from Sanchez-Rueda et al. (2019). Copyright 2019 RSC Pub. **(H)** Dendritic oligonucleotides on DNA brick nanostructures. Adapted with permission from Kim and Yin (2020). Copyright 2020 Wiley. **(I)** Albumin dendron conjugates for DNA origami coating. Adapted with permission from Auvinen et al. (2017). Copyright 2017 Wiley. **(J)** Dendritic alkyl decorating DNA nanocubes with high-affinity binding to human serum albumin. Adapted with permission from Lacroix et al. (2017). Copyright 2017 ACS.

Natural DNA backbone is composed of repeated deoxyribose and phosphate groups. It is the target of DNases which catalyze the hydrolytic cleavage of phosphodiester linkages. Chemical modification of the backbone can drastically enhance its biological stability by hindering the attack of nuclease. These backbone-modified nuclei acids containing unnatural components can be called synthetic nucleic acid polymers, which includes xeno-nucleic acids (XNA), peptide nucleic acids (PNA), locked nucleic acids (LNA), threose nucleic acids (TNA), and phosphorothioate DNA (Figure 2A) (Burns et al., 2013; Pedersen et al., 2015). These hybrid nanostructures function no less efficiently than their natural analog counterparts. On that basis, using a series of engineered polymerases, Philipp's group synthesized nano-objects fully composed of unnatural nucleic acids, where they used 2′F-RNA, 2′-fluoroarabino nucleic acids (FANA), hexitol nucleic acids (HNA), and cyclohexene nucleic acids (CeNA) to assemble tetrahedral structures (Figure 2B). HNA tetrahedrons remained intact after 8 day incubation in serum-containing cell culture media, while tetrahedrons of natural DNAs were fully degraded in 2 days (Taylor et al., 2016).

Kim K. R. et al. used L-DNA, a mirror form of natural D-DNA, as building materials for DNA nanocarriers (Figure 2B). L-DNA has identical thermodynamic properties to D-DNA, but it has significantly higher serum stability, prolonged *in vivo* residency, and enhanced cellular uptake. In xenograft mouse models, an L-DNA nanocarrier showed better effect on tumor inhibition than D-DNA tetrahedrons or PEGylated liposomes while loaded with doxorubicin (Kim K. R. et al., 2016).

### Covalent Crosslinking

Self-assembled DNA nanostructures generally contain several to thousands of DNA single strands, which impose a high density of nicking points that render the DNA structures vulnerable to cationic depletion and nuclease degradation. To circumvent this instability issue induced by nicking points, covalent crosslinking of DNA strands represents one elegant solution. For instance, crosslinking of DNA was shown to be able to stabilize DNA structures in the presence of denaturing agents or at elevated temperature (Rajendran et al., 2011). In 2015, Manetto reported a 6-helix bundle DNA structure composed of terminal functionalized single stranded DNA with linear alkyne and azide moieties. A copper-catalyzed click reaction was performed after assembly to bridge nicking points, resulting in the formation of cyclized strands interlinking with each other (Figure 2C). After crosslinking, the 6-helix bundles were able to remain intact in buffers lacking magnesium and stay resistant to exonuclease-I (Cassinelli et al., 2015). In 2018, Gerling et al. reported another strategy to achieve crosslinking of DNA origami structures without introducing chemical moieties (Figure 2D). It was accomplished by light-triggered formation of pyrimidine dimers between adjacent thymine or cytosine bases. These bases are designed to be placed in adjacent terminals of nicks formed by strands, branches, or helixes. Comparing with non-crosslinked structures, crosslinked origami objects needed a significantly prolonged time (~6-fold) to be degraded in biological media (Gerling et al., 2018).

### Encapsulation

Encapsulation of DNA structures by functional agents may also enhance their stability. For instance, Shih et al. encapsulated DNA origami structures via *in-situ* formation of liposomes surrounding the structures (Perrault and Shih, 2014). The lipid bilayer envelope not only shielded DNA nanocarriers from enzymes in serum, but also changed their surface characters which is important to determine immune responses and biodistributions. After lipid bilayers' encapsulation, immune activation by DNA structures was decreased by 2 orders of magnitude, and pharmacokinetic bioavailability was improved by a factor of 17 (Figure 2E).

Cationic polymer is another type of material used for DNA structure encapsulation. Cationic polymers can tightly adsorb onto the negatively charged phosphate backbone of DNA through electrostatic interaction. The molecular ratio of polymers to DNA structures is critical since an improper ratio may cause unwanted aggregation or distortion (Kiviaho et al., 2016). As a widely used anti-opsonization agent, polyethylene glycol (PEG) has shown to have broad application potentials in drug delivery. Introducing PEG into coating polymers is a common strategy to alleviate non-specific adsorption of proteins (Dai et al., 2014). Shih's group enveloped DNA barrels with oligolysine-PEG copolymers, which helped the DNA barrel to avoid rapid renal clearance and extended its blood half-life from 9 to 45 min (Figure 2F) (Ponnuswamy et al., 2017). Similar with synthetic polymers, a series of diblock recombinant proteins were also employed, which are composed of a non-specific DNA-binding domain (e.g., Sso7d, K12) and a hydrophilic unstructured peptide segment. The diblock proteins form brush-like structures around the DNA to provide better resistance to enzyme degradation (Figure 2G) (Sanchez-Rueda et al., 2019).

Inspired by spherical nucleic acids, of which the densely packed nucleic acids are highly resistant to nuclease degradation, Kim and Yin coated the outer surface of DNA brick nanostructures with dendritic oligonucleotides through base paring between overhang strands and oligonucleotides (Figure 2H) (Kim and Yin, 2020). This method can increase the biostability of DNA brick structures in comparison to their naked counterparts.

### Bound to Albumin

As the most abundant protein, albumin holds a relatively long circulation half-life in blood. Physiologically, albumin can bind with other molecules such as bilirubin, fatty acids, and metal ions to extend their circulation time and to improve their biodistribution. Albumin is also an anti-opsonization agent that can prevent phagocytosis of nanocarriers. Therefore, albumin is a promising agent to be used for drug delivery. In fact, some albumin-bound chemotherapeutics have already been approved by the FDA for clinical use, such as Abraxane, an albumin-bound form of Pacilitaxel.

However, it is generally challenging to achieve high affinity binding between albumin and DNA nanocarriers. To address this technical challenge, Kostiainen et al. engineered bovine serum albumin (BSA) into biohybrid macromolecules with cationic dendritic conjugates (polyamine analogs). The conjugate was anchored to BSA via a cysteine–maleimide bond with cysteine residues (Figure 2I). Thus, the engineered BSA can attach to DNA structures through electrostatic interactions between polyamine and DNA backbones (Auvinen et al., 2017). Lacroix et al. reported another method by decorating DNA structures with dendritic alkyl chains (Figure 2J). Tuning the number and orientation of the amphiphilic decorations enables DNA nanocubes to bind with human serum albumin (HSA) with a high affinity in low nanomolar range. Meanwhile, HSA did not hinder the activity of cargo antisense oligonucleotide *in vitro* (Lacroix et al., 2017). Since these two reports were lacking in *in vivo* experiments, the efficiency of albumin pre-binding strategy needs to be validated in animal models.

## Targeting Strategies *in vivo*

After systemic administration, another big hurdle ahead of DNA nanostructure carriers is the targeting delivery to specific organs/tissues of interest. Enabling selective accumulation of drug molecules in targeted sites not only boosts its therapeutic efficacy but also alleviates off-target delivery-related systemic toxicity. Therefore, targeting functionality is a pivotal factor determining the therapeutic performance of drug delivery systems. In this section, *in vivo* targeting strategies that have been used on DNA nanocarriers is summarized.

### *In vivo* Biodistribution of Pristine DNA Nanostructures

**Figure 3:**
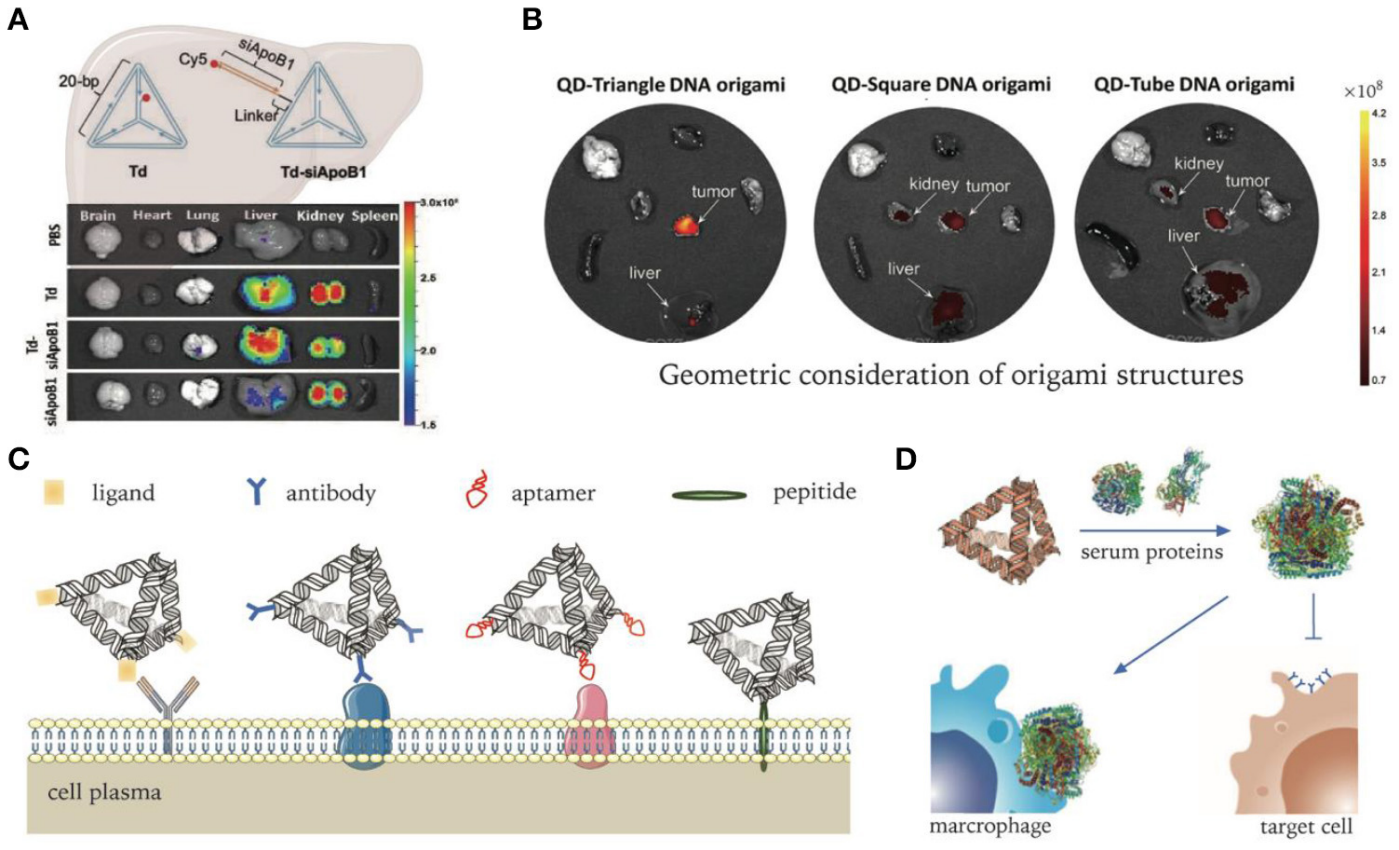
DNA nanocarriers targeting organs, tumors, and cells. **(A)** Delivery of ApoB1 siRNA to hepatic cells by pristine DNA tetrahedrons. Adapted with permission from Kim et al. (2020). Copyright 2020 RSC Pub. **(B)** DNA origami's passive delivery to malignancy. Adapted with permission from Zhang et al. (2014). Copyright 2014 ACS. **(C)** Modifications of DNA nanocarriers for active targeting to specific cells. **(D)** Non-specific protein adsorption to DNA nanocarriers in serum environment. Adapted with permission from Oh et al. (2018). Copyright 2018 Springer Nature.

In order to investigate the *in vivo* performance of DNA nanocarriers, numerous studies have firstly examined their biodistributions in various animal models. Generally, pristine DNA nanostructures have a preferential accumulation in organs like the liver, kidneys, and lymph nodes. For instance, as one of the simplest DNA structures, DNA tetrahedrons (Tds) have been widely researched. Kim K. R. et al. reported a tendency of hepatic accumulation of Tds after intravenous injection (Kim K. R. et al., 2016). By changing DNA backbones to increase biostability, the phenomenon of hepatic accumulation can be significantly enhanced. They harnessed this property of Td for liver delivery of siRNA successfully, which targets the overexpressed ApoB1 mRNA in hypercholesterolemia. ApoB1 siRNAs were loaded on Tds through DNA linkers extending from each side. As a result, an ~20–30% decrease in serum lipid levels was observed when compared with PBS controls (Figure 3A) (Kim et al., 2020). Jiang et al. studied the biodistribution of radiolabeled DNA origami nanostructures (DONs) through positron emission tomography (PET) imaging. DONs of three different geometries, including rectangle, triangle, and tube, were tested. All three exhibited predominant renal uptake, among which triangular DONs were used to effectively treat acute kidney injury in mice model (Jiang et al., 2018).

Apart from intravenous injection, subcutaneous injection is another commonly used administration route. The negative surface charge and size (6–10 nm) of Tds were found to be quite suitable for lymphatic drainage. Moreover, Tds can be easily uptake macrophages and have good intracellular stability, which extends their lymph node retention time. Taking advantages of these properties, Kim et al. labeled Tds with Cy5 fluorophore to visualize the sentinel lymph nodes of tumor (Kim et al., 2013). Compared with linear DNA probes, Tds showed enhanced translocation in lymph nodes and a prolonged retention time in mice xenograft models.

### Passive Delivery to Tumors

It is generally believed that most solid tumors exhibit enhanced permeability and retention (EPR) (Fang et al., 2011; Park-Windhol and D'Amore, 2016), though this has become controversial in recent years (Sindhwani et al., 2020). For DNA nanostructures, the impact of shape diversity on EPR effect has been revealed. Zhang et al. demonstrated that triangle-shaped DNA origami exhibited optimal tumor passive targeting accumulation compared to rectangular and tubular origami structures (Figure 3B) (Zhang et al., 2014). They revealed that triangle-shaped origami accumulated at the tumor site and reached peak levels at 6 h and maintained high levels for 24 h after intravenous administration. Kim et al. built a library of wireframe DNA objects of various backbones for *in vivo* screening (Kim et al., 2019). According to their results, cages with backbone modifications had better tumor accumulation. In terms of shape effect, pyramid-shaped nanocages exhibited the highest tumor specific delivery efficiency.

### Active Delivery to Cells

With the discovery of pathological mechanisms, disease-specific molecular markers or microenvironmental characteristics are constantly revealed. Cells harboring pathological changes, such as cancerization or infection, usually over-express certain molecular receptors or biomarkers which could well serve as targets for active delivery. In addition, microenvironmental parameters like pH or oxygen level may significantly change under pathological conditions that may be targeted to realize active delivery (Salahpour Anarjan, 2019).

Molecular ligands used for targeting delivery include antibodies, aptamers, receptor ligands, functional peptides, etc. They can be tethered onto DNA nanocarriers via various conjugation methods to realize targeting functionality (Figure 3C) (Wu et al., 2013; Setyawati et al., 2016; Xia et al., 2016; Santi et al., 2017). Active targeting functionalized DNA nanocarriers have been demonstrated to enhance brain permeability, which passive delivery fails to reach due to the existence of blood–brain barrier (BBB). Tian et al. modified framework tetrahedral DNA nanoprobes with peptides targeting both brain capillary endothelial cells and malignant glioma cells. Through receptor-mediated transcytosis, DNA nanoprobes successfully passed through the BBB model and then entered the cytoplasm of the tumor cells (Tian et al., 2018).

However, despite pronounced selectivity *in vitro*, some ligands were found to lose targeting ability *in vivo* (e.g., transferrin) (Mirshafiee et al., 2013). One important reason for the failure of the targeting capability in complex biological milieu is the occurrence of biotransformation when adsorbed by serum proteins, which not only abolishes molecular recognition capability, but also induces clearance by phagocytes (Figure 3D) (Oh et al., 2018). Therefore, systematic study and optimization need to be conducted to fully realize the targeting capability of various ligands for *in vivo* delivery applications.

## Cell Entry Routes and Intracellular Fate

**Figure 4:**
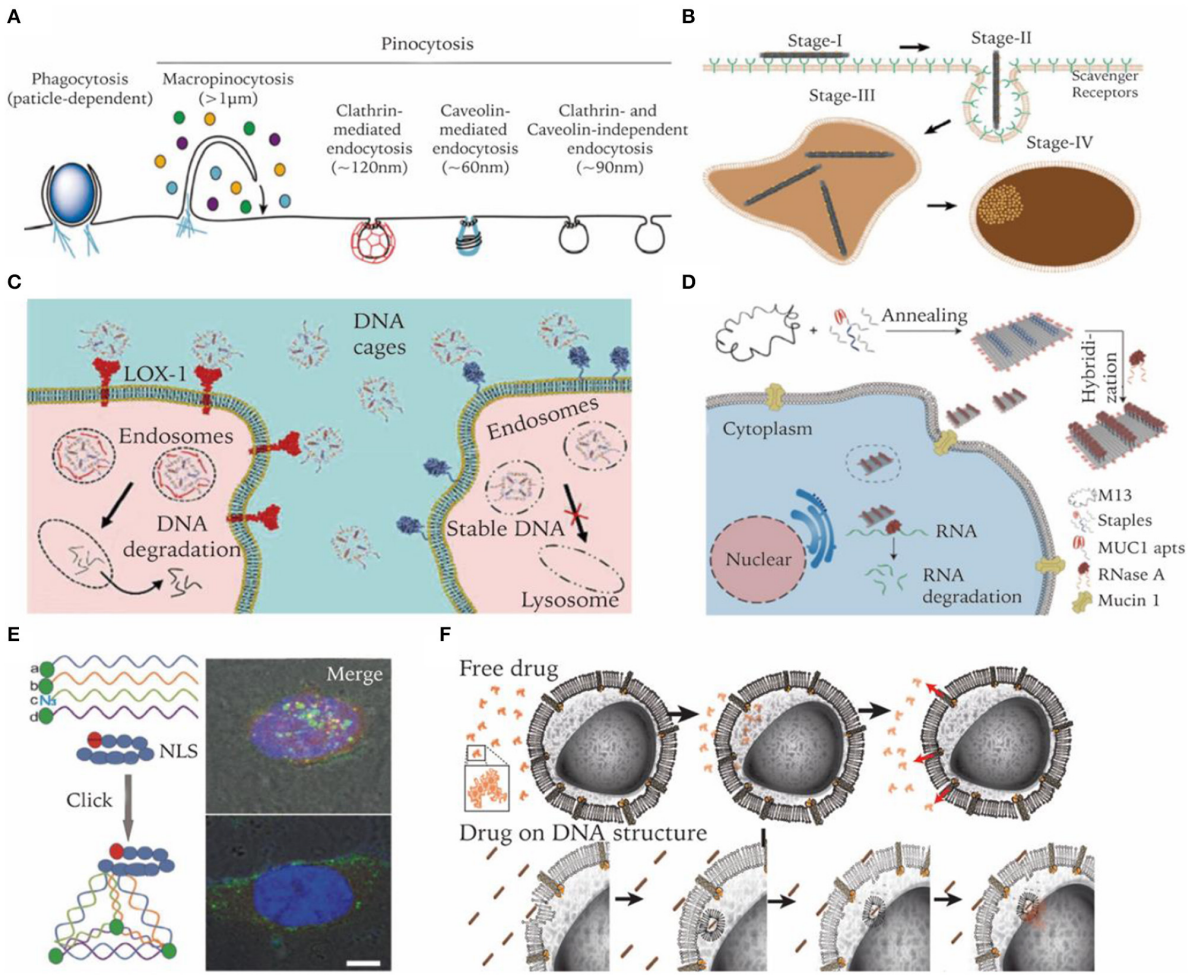
Enhancing cell entry of DNA nanostructures and regulating their intracellular fate. **(A)** Endocytosis pathways involved in nanoparticle's cell uptake. Adapted with permission from Lee et al. (2016). Copyright 2016 RSC Pub. **(B)** Cell internalization process of a DNA origami rod structure. Adapted with permission from Wang et al. (2018). Copyright 2018 ACS. **(C)** Bypass lysosome pathway through targeting specific receptors. Adapted with permission from Raniolo et al. (2018). Copyright 2018 RSC Pub. **(D)** Lysosome escape and delivery of active enzymes by DNA origami modified with MUC1 aptamer. Adapted with permission from Zhao S. et al. (2019). Copyright 2019 ACS. **(E)** Lysosome escape of DNA tetrahedrons modified with nucleus-targeting signaling peptides. Adapted with permission from Liang et al. (2014). Copyright 2014 Wiley. **(F)** Drugs on DNA nanocarriers circumvent efflux-pump-mediated drug resistance mechanisms. Adapted with permission from Halley et al. (2016). Copyright 2016 Wiley.

Over billions of years of evolution, the cell membrane has become a wall to protect sophisticated cellular organelles from the extracellular environment. Only selected substances can pass through the membrane. Most nanoscale substances, including molecules and nanoparticles, enter cells through an energy consuming pathway called endocytosis, which can be categorized into several distinct subtypes (Figure 4A) (Lee et al., 2016). Phagocytosis primarily exists in immune cells like macrophages to engulf large particles (>0.5 μm). Pinocytosis is widely adopted by all types of cells, which can be further subdivided into macropinocytosis, clathrin-mediated endocytosis, caveolae-mediated endocytosis, clathrin-, and caveolae-independent endocytosis. After endocytosis, nanocarriers are encapsulated and transported in membrane vesicles like endosomes (Conner and Schmid, 2003). Proteins on the endosome membranes induce the maturation of endosomes into lysosomes and then transport cargo into corresponding subcellular regions. The permeability of lysosomal membranes is complicatedly regulated and plays an important role in determining the intracellular fate of nanocarriers (Johansson et al., 2010). Thus, it is critical to tune and to optimize DNA nanocarriers in order to achieve potent intracellular delivery performance.

### Enhancing Cellular Uptake Efficiency

Since DNA and RNA molecules are negatively charged due to phosphate groups on the backbone, electrostatic repulsion from cell membranes makes it difficult for them to enter cells efficiently. As mentioned earlier, encapsulating DNA nanocarriers with cationic polymers can facilitate its cell uptake. For instance, Lee J. B. et al. used polyethylenimine (PEI) to condense sponge-like structures generated from RCA reactions, which significantly enhanced the cell uptake efficiency of siRNA sponges and achieved significant knockdown of target mRNA (Lee J. B. et al., 2012).

Despite its anionic nature, DNA nanostructures of prescribed three-dimensional geometries likes cages or origami structures were found to be able to enter cells efficiently without the aid of transfection reagents. Ligand binding receptors on cell membranes are responsible for the uptake of DNA nanostructures. Liang et al. demonstrated that DNA tetrahedrons were internalized by a caveolin-dependent endocytosis pathway and transported to lysosomes in a microtubule-dependent manner (Liang et al., 2014). Vindigni et al. reported that scavenger receptor LOX-1 was responsible for mediating pristine DNA nanocages into cells. Cells overexpressing LOX-1 internalized cages 30 times higher than their low-expressing counterparts (Vindigni et al., 2016). Wang et al. reported that cancer cells can readily uptake DNA origami nanostructures (DONs) with various sizes and shapes with high efficiency. Further study revealed that DONs of a larger size and higher aspect ratio had increased uptake efficiency compared to their counterparts. Moreover, they visualized the multi-stage internalization process of DONs on a high-resolution single particle level (Figure 4B) (Wang et al., 2018). Lee et al. reported that the number and disposition of targeting ligands (i.e., folate) on the DNA tetrahedral nanocarriers largely affect its *in vitro* and *in vivo* delivery efficiency (Lee H. et al., 2012).

### Modulating Intracellular Fate

Biomolecular drugs like ASOs and siRNAs are prone to enzymatic degradation. After entering cells through endocytosis, they need to escape from lysosomes to cytoplasm or avoid endosomal encapsulation in the first place. Strategies like membrane fusion and acid swelling have been applied in many delivery systems to circumvent this challenge (Smith et al., 2019), which, however, cannot be easily adapted by DNA nanocarriers. A more feasible strategy for DNA nanocarriers would be trying to avoid endolysosomal entrapment. It was reported that endosomes induced by certain receptors of caveolae-mediated endocytosis may transport to non-lysosome organells (Parton and Simons, 2007). Targeting these receptors is a practical method for DNA nanocarriers avoiding degradation in lysosome. Folic acid, albumin, cholesterol, and transferrin are ligands that have been proven to be able to serve this purpose (Figure 4C) (Mirshafiee et al., 2013). Some aptamers have also been reported to be able to guide DNA nanocarriers to avoid lysosomal degradation. For example, nucleolin-targeting aptamer AS1411 can be internalized into a wide variety of cancer cells and migrates to the nucleus. It was internalized via macropinocytosis in cancer cells, but via a non-macropinocytic pathway in normal cells (Reyes-Reyes et al., 2010). Charoenphol et al. incorporated AS1411 aptamers into DNA pyramids, which led to selective inhibition of cancer cells (Charoenphol and Bermudez, 2014). MUC1 aptamer is another example. In cancer cells, MUC1 protein was reported to be crucial for the stabilization of lysosome membranes (Dudeja et al., 2007). Zhao S. et al. found that MUC1 aptamer-tethered DNA origami structures can be efficiently transported to cytoplasm within 24 h. In contrast, naked DNA origami structures were trapped in lysosomes (Figure 4D) (Zhao S. et al., 2019). Fan et al. reported another strategy of escaping lysosomes by using peptides. They functionalized the tetrahedral DNA nanostructures with nucleus-targeting signaling (NLS) peptides through a click reaction. Though entering cells by the endocytosis route, NLS modified structures were transported to the nucleus after 16 h and remained intact (Figure 4E) (Liang et al., 2014).

In certain scenarios, lysosomal entrapment of nanocarriers may not necessarily be a disadvantage. For instance, in some cases, drugs need to be localized to endosomes in order to function. Castro et al. used rod-shaped DNA origami to load and deliver daunorubicin to cancer cells to circumvent efflux-pump-mediated drug resistance. Compared to free drugs entering cells via passive diffusion, delivery of daunorubicin by DNA origami carriers led to a higher amount of drug accumulation and better resistance to pump-mediated drug efflux (Figure 4F) (Halley et al., 2016). The endosomatic pathway also plays important roles in regulating innate immune responses. DNA structures trapped in endosomes are more accessible by specialized receptors of the innate immune system. Schüller et al. reported that DNA origami rods decorated with CpG-containing oligonucleotides are quite efficient for cellular immunostimulation. They can trigger stronger innate immune responses than a standard carrier system such as lipofectamine (Schüller et al., 2011).

## DNA Nanocarriers Responding to Microenvironments

**Figure 5:**
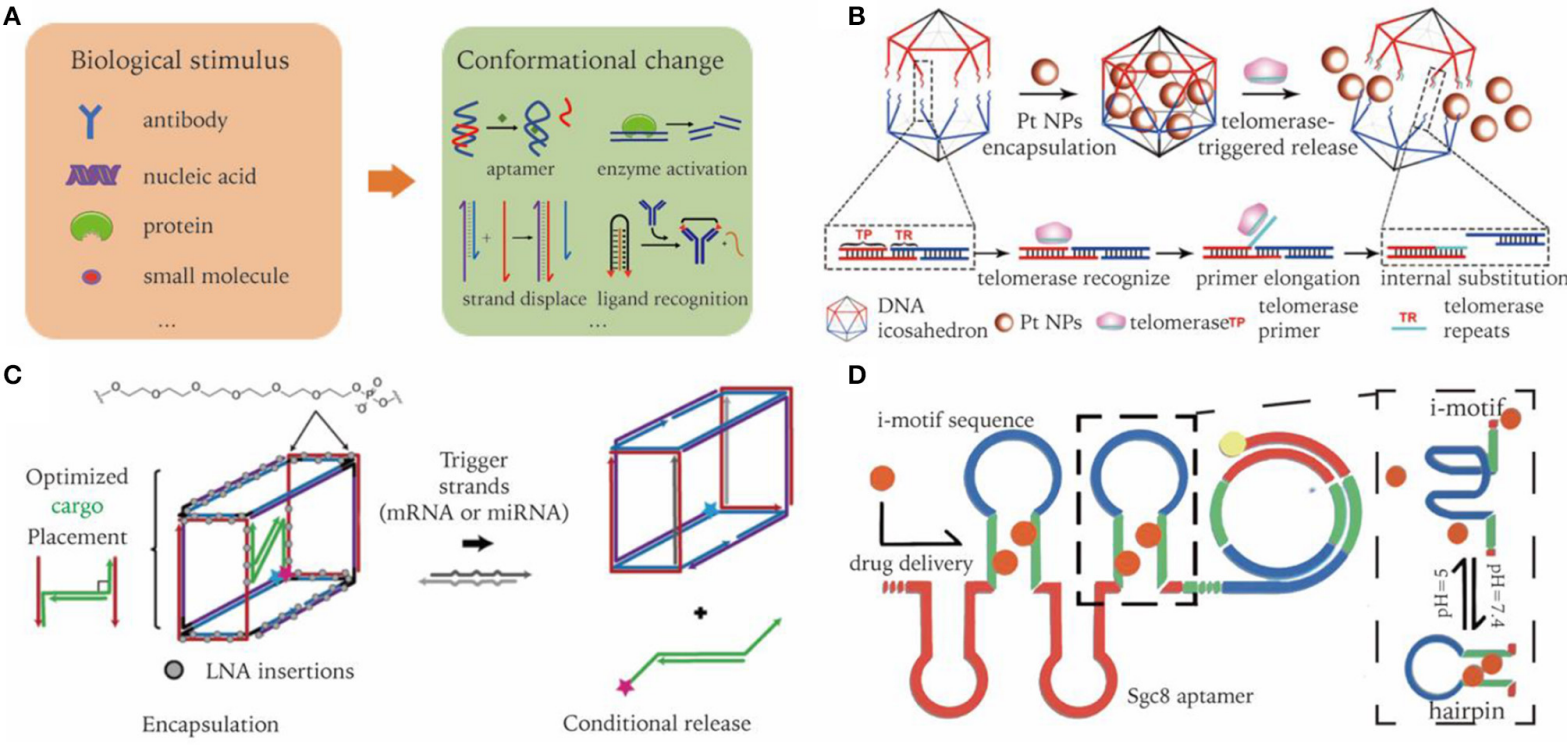
Dynamic DNA nanocarriers for conditional triggered release of cargos in biological environment. **(A)** Biological inputs and reconfigurable elements to achieve stimuli responsive cargo release. **(B)** Active telomerase triggers detachment of DNA icosahedron nanocages resulting in targeted release of drugs. Adapted with permission from Ma et al. (2018). Copyright 2018 Wiley. **(C)** DNA nano-suitcases conditionally release siRNA cargos in the presence of oligonucleotide triggers. Adapted with permission from Bujold et al. (2016). Copyright 2016 ACS. **(D)** RCA products incorporated with pH responsive i-motif sequences. Adapted with permission from Zhao et al. (2018). Copyright 2018 ACS.

Microenvironments of pathological sites generally exhibit distinct characteristics that are different to healthy sites, e.g., acidic and hypoxic conditions of tumor microenvironments, or overexpression of certain molecular biomarkers. Such differences in environmental characteristics could serve as another type of target for nanocarriers to aim for. In order to arm pristine DNA nanostructures with stimuli responsive capability, dynamic elements need to be integrated into the designed nanocarrier (Figure 5A) (Harroun et al., 2018; Zhang Y. et al., 2019). In the following section, we discuss some mechanisms that may be incorporated into DNA nanocarriers to realize environmental responsive cargo delivery.

### Aptamer-Enabled Robotic Systems

Aptamers are single-stranded nucleic acids of a specific sequence that can selectively recognize and bind to a target (e.g., ions, molecules, proteins), which are generally acquired from multiple rounds of *in vitro* selection (Shangguan et al., 2006). To enable target-binding induced conformational change, a duplexed aptamer is designed by using a complementary strand to hybridize to a specific portion of the aptamer sequence, which may be released from the aptamer once the target of high affinity is present (Munzar et al., 2019). This element has been used as a key to unlock DNA origami containers to realize cell specific cargo transportation (Douglas et al., 2012). Recently, Li et al. reported the successful *in vivo* application of an autonomous DNA robot as an responsive drug delivery system (Li et al., 2018). This robotic container was fastened by aptamer AS1411, which can bind to nucleolin that overexpressed on tumor-associated vascular endothelial cells in a tumor microenvironment. Upon binding to nucleolin, the DNA robot opens up to unload its cargo thrombin to induce thrombosis in tumor-associated blood vessels to starve and eventually kill tumor cells.

### Enzyme Mediated Cargo Release

Nuclease degradation of DNA nanocarriers might represent the simplest way to induce cargo release (Sun et al., 2014). Nevertheless, it is limited to the delivery of nuclease-resistant chemical drugs. Different to nuclease, enzymes like telomerase can recognize specific DNA sequences. Integrating these sequences into DNA nanostructures enables them to selectively interact with enzymes (Yin et al., 2004). Ma et al. reported a telomerase-responsive DNA-icosahedron nanocarrier that can selectively release caged platinum-nanodrugs into cisplatin resistance cancer cells. Telomerase primer sequences were incorporated into the edges of DNA icosahedrons, serving as a recognition element of telomerase. After binding with DNA icosahedrons, telomerase was activated and started to generate telomeric repeats, which hybridize with downstream toehold sequence, resulting in the detaching of drugs (Ma et al., 2018). (Figure 5B).

### Strand Displacement Induced Cargo Unloading

Strand displacement is widely used for constructing dynamic structures, in which one strand in a DNA duplex can be released and replaced by another strand through a toehold design (Yurke et al., 2000). The invader strand can be mRNA, miRNA, etc. The released strand may then serve as a trigger of subsequent cascade reactions. In terms of drug delivery, released strands typically have a therapeutic effect, such as the delivery of siRNA or ASOs. For instance, Katherine et al. designed a DNA nano-suitcase that encapsulated a siRNA cargo, which can be specifically released upon recognition of an oligonucleotide trigger. The siRNA cargos partially bind to the edge of DNA cages, with the leftover sequence serving as a toehold for strand displacement. They showed that the whole construct was assembled in high yield, with cargo released on demand, and remained intact in biological conditions for a long period of time (Figure 5C) (Bujold et al., 2016).

### pH Responsive Cargo Release

DNA triplex and i-motif are two commonly used pH-responsive structures. They follow strict sequence requirements, which takes advantage of binding equilibrium shifts according to the A^+^-C base pair formation in acidic solution (Fu et al., 2019). An acidic extracellular microenvironment is well-recognized in the process of oncogenesis. Zhao et al. incorporated i-motif into RCA products with bilateral complementary sequences. In an acidic tumor environment, the i-motif sequences at the loop region fold, resulting in the opening of the stem region. Thus, previous intercalated drugs are released in response to environmental pH. Functionalized with aptamers, the resultant drug-carrier system accomplishes *in vivo* targeted delivery, and pH-stimulated sustained release of Dox (Figure 5D) (Zhao et al., 2018).

## Challenges and Opportunities For Clinical Applications

The unique advantages of using DNA nanocarriers as drug delivery systems is quite obvious. First, the size and shape of DNA nanocarriers can be finely tuned. And the robust assembly of DNA can greatly alleviate batch-to-batch variations. Second, the numbers and positions of ligand modifications or drug loadings on DNA nanocarriers may be precisely programmed. Third, although the biological environment is quite different from where DNA nanocarriers are originally assembled, optimizing their biostability, circulation time, biodistribution, cell entry routes, and intracellular fate enable DNA nanocarriers to perform well *in vivo*. Finally, various practicable modifications on DNA allow the integration of DNA nanostructures with other materials, such as AuNPs and liposomes (Sun and Gang, 2011; Yang et al., 2016), making DNA nanocarriers a robust system with a large potential of holding multiple functionalities.

In laboratory settings, DNA nanocarriers have demonstrated the capability to conquer drug resistance of cancer cells, serve as an efficient non-viral vector for gene therapy, induce target thrombosis in tumor vessels, improve the restoration of kidney functions, and visualize sentinel lymph nodes. These problems center around demanding better solutions within real clinical settings. However, there is still a long way to go to translate their good performance in cells or in mice to clinical application in patients. More studies need to be conducted to better understand the determining factors of *in vivo* performance. It remains unclear how the physical and chemical properties, such as surface charges, oligonucleotide modifications, or protein adhesions of DNA nanostructures, affect the pharmacokinetic bioavailability. The preference of hepatic and renal uptake of DNA nanocarriers limits their applications in other organs. At a cellular level, cell entry through the endocytosis pathway is far from highly efficient and selective. Production cost is another concern, especially when chemical modifications are in high demand. At last, although DNA material is biodegradable, comprehensive assessments of their biosafety in humans are essential prior to real clinical applications. With these issues appropriately addressed, we believe DNA-based nanocarriers hold a bright future toward serving as potent drug delivery systems for treating many diseases in clinic.

## Author Contributions

All authors listed have made a substantial, direct and intellectual contribution to the work, and approved it for publication.

## Conflict of Interest

The authors declare that the research was conducted in the absence of any commercial or financial relationships that could be construed as a potential conflict of interest.
